# Mutation bias and adaptation in bacteria

**DOI:** 10.1099/mic.0.001404

**Published:** 2023-11-09

**Authors:** James S. Horton, Tiffany B. Taylor

**Affiliations:** ^1^​ Milner Centre for Evolution, Department of Life Sciences, University of Bath, BA2 7AY, UK

**Keywords:** mutation bias, adaptation, bacterial evolution, mutation hotspot, stress response, evolutionary forecasting

## Abstract

Genetic mutation, which provides the raw material for evolutionary adaptation, is largely a stochastic force. However, there is ample evidence showing that mutations can also exhibit strong biases, with some mutation types and certain genomic positions mutating more often than others. It is becoming increasingly clear that mutational bias can play a role in determining adaptive outcomes in bacteria in both the laboratory and the clinic. As such, understanding the causes and consequences of mutation bias can help microbiologists to anticipate and predict adaptive outcomes. In this review, we provide an overview of the mechanisms and features of the bacterial genome that cause mutational biases to occur. We then describe the environmental triggers that drive these mechanisms to be more potent and outline the adaptive scenarios where mutation bias can synergize with natural selection to define evolutionary outcomes. We conclude by describing how understanding mutagenic genomic features can help microbiologists predict areas sensitive to mutational bias, and finish by outlining future work that will help us achieve more accurate evolutionary forecasts.

## Introduction

Each bacterial genome boasts an arsenal of genes that provide robustness against changing environments, and bacterial cells can acquire access to yet more phenotypes through the horizontal transfer of genetic material [1]. However, when exposed to new selective challenges, novel adaptive phenotypes are often generated through the process of mutation. With typically large population sizes and rapid generational times, bacterial populations are hotbeds for adaptation through mutation, allowing them to rapidly evade extinction against numerous threats.

The adaptive capability of microbes through mutation is well documented and well understood – it is a key reason why we struggle to eradicate pandemics [2] and why antimicrobial resistance is such a potent threat [3]. As such, many researchers are dedicated to elucidating the consequences of mutation, such as how gene regulatory networks are re-organized and how functions of protein products are altered following a mutational change. Complementary to these efforts is the study of the mutation process itself. Because if we can understand the causes of mutations, we will be able to anticipate their arrival. This will provide us with the ability to form accurate short-term evolutionary forecasts – allowing us to be proactively poised to deal with the consequences of mutation as soon as they occur.

Mutation is the primary means of generating genetic variation and is often described as a stochastic force. This means that the process of mutation relies on chance events. However, chance does not mean all mutations are equally likely to happen. Instead, mutations are biased. They occur at different rates, with certain types of mutations being more common than others (e.g. a transition substitution bias [4]), and certain locations in the genome being more mutable than others (e.g. those farther from the replication origin [5]). As such the raw material that generates genetic variation and facilitates adaptation is provided unevenly, with certain mutations appearing in the population more often than others [6]. These effects, while commonly overlooked, can have profound impacts on the course of adaptation and evolution.

The relationship between mutation bias and bacterial adaptation can sometimes be antagonistic, as the types of mutations that are selectively beneficial may be in direct conflict with those promoted by mutation bias. For example, bacterial genomes may universally be biased to mutate from GC → AT, and yet some bacterial genomes continue to be GC-rich [7]. Similarly in viruses, Rice *et al*. found that the SARS-CoV-2 genome exhibits selection against uracil content despite changes to uracil being promoted by mutation bias [8]. However, when mutation rates are higher at a nucleotide position that can confer an adaptive advantage, mutation bias can synergize with selection to make certain outcomes more likely than others. These mutational biases can be incredibly local and specific, with adaptive outcomes being defined by localized mutational ‘hotspots’ within single genes [9]. Cano *et al*. have recently shown that known mutational biases are reflected in adaptive outcomes for *Saccharomyces cerevisiae*, *Escherichia coli* and *Mycobacterium tuberculosis* [10]. This study highlights that the uneven production of mutations owing to bias can synergize with selection and help to steer the course of evolution.

With recent research revealing the impact of mutation bias on adaptation within the bacterial domain [10, 11], there is an opportunity for microbiologists from various disciplines to consider what role mutation bias plays in the evolution of their organism(s) of interest. In this review, we will describe the features that cause mutation bias in bacterial genomes and discuss how we can use this knowledge to identify areas more prone to mutation. We will also outline the contexts when bias can be a defining factor in bacterial adaptation and highlight how the evolution of pathogenic microbes in the clinic are susceptible to agents of mutation bias. We will conclude by discussing future research avenues that will facilitate the discovery and consequence of bias throughout the bacterial genome.

## The heart of the matter – local and genome-wide causes of mutation bias

**Fig. 1.**
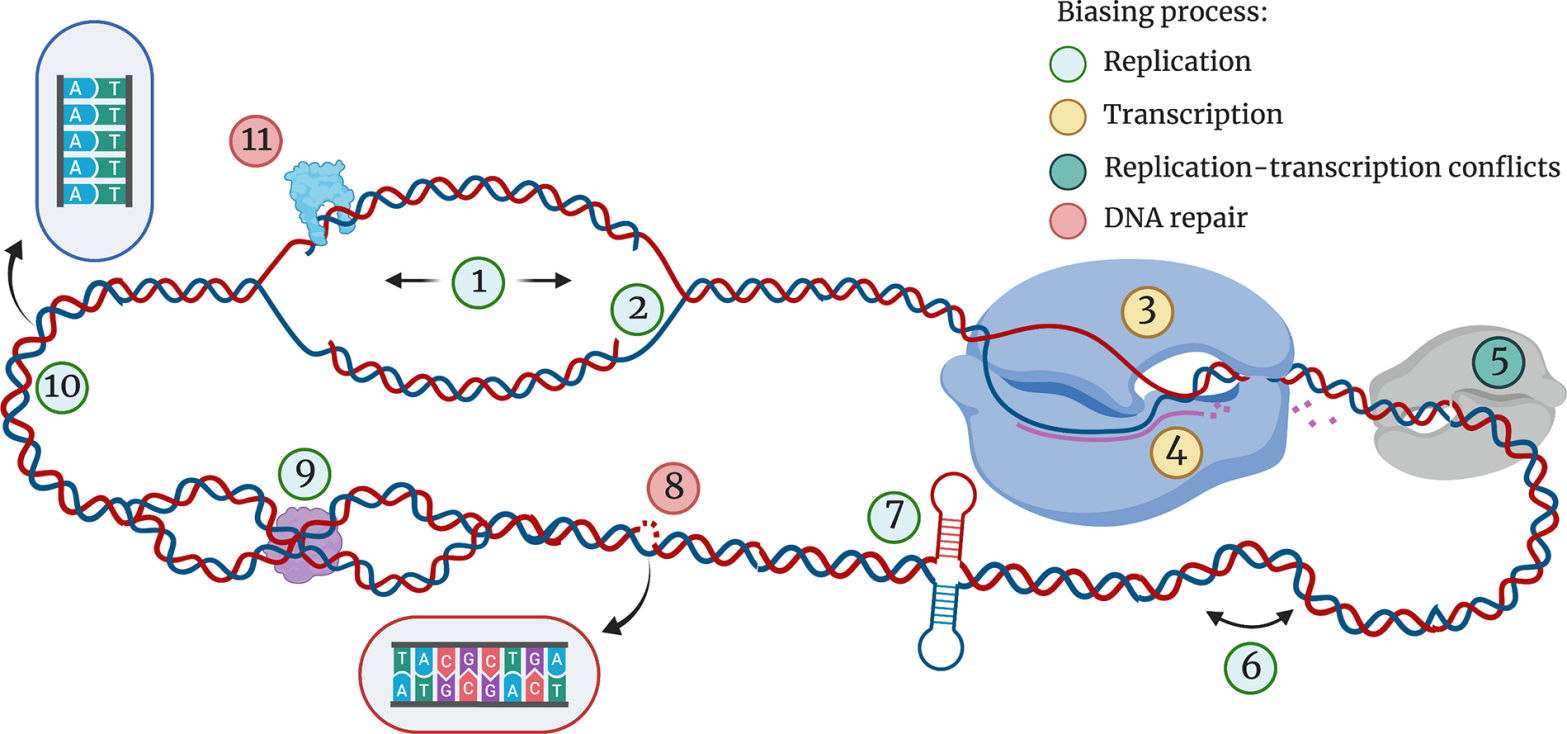
Features affecting regional and localized mutation bias throughout the bacterial genome. Mutation bias primarily results from replication [157], transcription [158], collisions between replication and transcription complexes [159], and repair enzyme activity [160], as denoted by the key. Labelled features are described per the following format: *n. Mechanism that causes mutation bias. Location in the genome impacted by mechanism*. 1. DNA polymerase stalling. Affected by genomic position/replication timing; polymerase fidelity is typically higher close to OriC [5, 24–26]. 2–3. Cytosine deamination. 2. More frequent on lagging ssDNA strand [28]. 3. More frequent on non-transcribed ssDNA strand [34–36]. 4. Transcription-coupled repair efficacy. Higher on transcribed strand [37]. 5. Head-on collisions between RNA polymerase and the replisome. Occur at genes encoded on the lagging strand [161–163], however the extent to which these events drive mutation is contested [41–43, 45]. 6. Deletion following recombination events. Occur at homologous direct repeats [114–117]. 7. Hairpin or cruciform secondary DNA structures. Formed at palindromic and quasi-palindromic sequences [109–112]. 8. Mismatch repair efficacy. Affected by nucleotide triplet of target sequence [5, 29, 44, 54]. 9. Nucleoid-associated proteins. DNA with high superhelical density may be more mutable during replication [20, 59, 60] but bound DNA is likely less sensitive to homologous recombination [58] and small mutagenic compounds [20]. 10. DNA polymerase slippage. Occur more frequently at homopolymer tracts [12, 29, 113]. 11. Presence of repair enzymes. Absence of mismatch repair generates ‘mutator’ alleles that often cause transition biases [5, 118, 119]; presence of repair enzymes increases mutagenesis at alternate non-canonical DNA secondary structures [164]. Figure created with BioRender.com.

Throughout this work we will often use the terms mutation rate, mutation bias and mutational hotspot. It is worth describing these terms first in more detail. Mutation rate is used to describe mutation in absolute, discrete terms. For example, *P. aeruginosa* has been quantified to have a mutation rate of 9.30×10^−11^ per nucleotide position per generation [12, 13]. This description provides the average genomic rate of mutation and therefore allows us to calculate the expected number of genomic mutations over time with consideration to population size. The genomic mutation rate multiplied by the number of replicating genomes in a population determines how often mutations are expected to occur, which is described as mutation supply. Mutation bias describes the relative differences in mutation rates between positions or mutation types. For example, a null assumption is that transversion mutations should occur at a ratio of 2 : 1 versus transitions, because 8/12 of the possible base substitutions are transversions. (This is an over-simplification [14], but suits our purposes for this example.) But when we see that transitions are more common than this in sequencing data, and we have controlled for the effects of selection [14, 15], we can state that there is a mutation bias toward transitions as they are relatively over-represented [14]. When considering adaptation, mutation bias details instances when the list of mutational targets (the total pool of mutations capable of producing a phenotype of interest) is skewed to produce certain mutations more than would be expected by chance. Biases mean that rates of mutation fluctuate throughout the genome, resulting in mutational coldspots that mutate less than the genome average, and mutational hotspots that are typically used to describe positions that mutate at much higher rates than the genome average [16–19]. Mutation rates are the product of many interacting mechanisms operating in tandem (Fig. 1), of which each introduces its own bias. Therefore, if we observe a change in mutation rate due to a mutation or an environmental trigger, this inevitably incurs a change in bias because not all mutagenic mechanisms will be equally affected.

Mutation is the result of chance mistakes that happen as genetic material is being replicated, transcribed, recombined and repaired (Fig. 1). The DNA–protein interactions driving these mutagenic mechanisms are therefore the harbingers of change. In its inactive or dormant state DNA exists as a stable, canonical duplex, which can also be modified by epigenomic features that help to protect DNA against environmental damage [20]. However, DNA does not perpetually exist in a dormant state. It is constantly unravelled and unwound to be used as a template for gene expression and to create copies of itself. These modifications expose DNA – they open the duplex into single strands, upon which toxins in the environment such as radicals and radiation can cause damage to the vulnerable sequence [21, 22].

Many mutational mechanisms are affected by the process of replication (Fig. 1). Bacteria possess a single origin of replication (OriC) and replicate DNA bi-directionally (theta replication) symmetrically outward from the origin to the replication terminus. Not only do bacterial genomes have a designated start and end point for replication, they also have strand-associated patterns where the leading strand and lagging strand are processed differently, with the leading strand copied continuously and the lagging strand copied in segments [23]. Both replication timing and the replicated strand are associated with mutation bias (Fig. 1). The fidelity of replication has been documented to be higher closer to the replication origin in *P. fluorescens *, *E. coli *, *S. enterica*, *V. fischeri* and *V. cholerae* [5, 24–26]. Further, lagging strand synthesis has been associated with higher fidelity in *E. coli* [27], but the lagging strand has also been observed to be more vulnerable to cytosine deamination [28]. Therefore, mutations can be disproportionally generated across large swathes of the genome, with mutation rates increasing around a megabase from the replication origin and changing depending on replicative strand.

While genome organization and strandedness can cause mutation rates to differentiate throughout the genome, mutational biases can fluctuate on a local level due to interactions between the replication complex and the local nucleotide context. For example, mononucleotide repeats (e.g. CCCCC) can cause the polymerase to slip and generate an insertion or deletion (indel) [12, 16, 29], while inverse repeat sequences (e.g. ATCGAC-n-GTCGAT) cause the generation of secondary structures that cause the polymerase to stall and produce a polymorphism [30] or double-stranded break [31]. The local nucleotide neighbourhood will often affect replication fidelity disproportionally depending on which strand the nucleotides are situated on. Homopolymeric tracts of guanine cause substitution mutations more frequently when on the leading strand [17], and mutations from inverse repeat DNA hairpins may more readily form on the lagging strand as it exists as a single strand for longer [23, 32]. However, more recent research has found that long inverted repeats can form hairpins on both strands, forming instead what is called a cruciform structure [31]. Local nucleotide context has been implicated in many mutational biases operating over small areas (e.g. small indels and single nucleotide polymorphisms) and so has been described as the major determinant of biases in bacterial systems [33].

Transcription is the other major process resulting in transient single-stranded DNA. Like replication, the two DNA strands are processed differently during transcription. RNA polymerase copies from one strand only – the transcribed strand – while the non-transcribed strand remains unbound as free single-stranded DNA during this process. As a result, the non-transcribed strand is more vulnerable to cytosine deamination [34–36]. Similarly, components of nucleotide excision repair, which correct damage endured during transcription, operate alongside RNA polymerase and so repair with higher fidelity on the transcribed strand [37, 38].

As transcription does not stop during periods of replication, there is a possibility that the two polymerase complexes can collide. If a gene is oriented co-directionally with replication, then DNA polymerase and RNA polymerase will process DNA in the same direction. Most genes, including most essential genes, are transcribed co-directionally with the replisome [39]. However as the replisome progresses faster than the RNA polymerase complex, co-directional collisions can still occur and cause genome instability [40]. Genes that are transcribed antagonistically to the replisome instead result in head-on collisions between the polymerase complexes [39]. This causes more potent genome instability, and head-on genes have been found to exhibit increased mutation rates in *B. subtilis* [41].

However, it remains unclear to what extent head-on collisions act as sources of spontaneous mutagenesis. Studies on *B. subtilis* and *E. coli* have found that patterns showing increased mutation in head-on genes can instead be explained by local nucleotide features that cause bias [42, 43]. Schroeder *et al*. found that nucleotide composition bias, i.e. that some nucleotides are found more on one strand than the other, can explain the increased rate of base substitutions found in head-on genes in *B. subtilis* [43]. (A rebuttal study however argued that this was only the case in laboratory evolved strains and did not explain substitutions in natural populations [44].) Similarly, Foster *et al*. observed that a subset of antagonistically oriented tRNA genes contained mononucleotide runs of length 5–8 bp, which are hotspots for indels, and this explained the overall enrichment for indels in head-on genes in *E. coli* [42]. The same study also found no positive correlation between rates of mutation in head-on genes and levels of expression, which would increase the opportunity for collision [42]. Sankar *et al*. designed an experimental system to contrast head-on and co-directional mutagenesis in *B. subtilis* [45]. They introduced the *thyP3* gene in both orientations with respect to replisome progression and placed the gene under control of an inducible promoter. They then contrasted the rate and spectrum of mutations observed in *thyP3* using an assay that captured loss-of-function mutations in the gene. Indel mutations were enriched at the promoter sequence and 5′ half of the gene when it was co-directional with replication and enriched at the 3′ half of the gene when in the head-on orientation. However, neither orientation nor induction of transcription affected base substitution rates in the coding region, but the head-on orientation did increase substitution rates in the promoter [45]. Overall, while head-on genes cause genomic instability in bacteria and may increase the rate of indel mutations and *cis*-regulatory substitutions, it is unclear whether they are a potent mutagenic force [39].

The final major source of mutational bias comes from DNA repair. Mismatch repair, which repairs errors incurred during replication, is a prominent enforcer of potent biases. Mismatch proteins or their equivalents are ubiquitous in bacteria [46]. Yet mutator variants, which are defective for mismatch repair, often evolve in bacterial pathogens that are under strong selection for novel adaptive mutations [47]. These include *S. enterica* pathogens [48], uropathogenic *E. coli* isolates [49] and strains of *P. aeruginosa* evolving in the cystic fibrosis lung [50]. Mutators have been widely reported to showcase strong biases. In the case of the highly conserved repair enzymes *mutS* and *mutL*, their inactivation drives a sharp increase in the rate of transition mutations and small indels [51]. In contrast, *E. coli* mutants defective in *mutT* or *mutY,* which remove oxidated guanine (8-oxo-G) that cause G:A mispairs [51], instead exhibit a bias toward A:T → C:G and C:G → A:T transversions, respectively [52, 53].

There is also a relationship between mutation frequencies found in mutator lines and local nucleotide sequence. Different rates of mutation have been observed depending on the nucleotide triplet, where the rate of mutation of a focal nucleotide varies depending on its immediate nucleotide neighbours [54]. While the most mutable nucleotide triplet can differ depending on the species, this trend has been reported for numerous mutator lines including in *B. subtilis* [44], *E. coli* [29], *P. fluorescens* [5] and *P. aeruginosa* [12]. Foster *et al*. highlighted that mismatch repair efficiency in *E. coli* can fluctuate by around 43-fold depending on the nucleotide triplet, showing that the fidelity of mismatch repair enzymes is affected by local nucleotide sequence [29]. Therefore, the major sources of mutational biases in the genome are driven by the interaction between the local nucleotide substrate and the protein complex interacting with it, whether that may be the replication complex, the transcription complex or repair enzymes.

## Setting the scene – drivers of mutation bias outside the bacterial genome

So far we have examined the DNA–protein interactions operating over local and broad genomic regions that cause elevations in mutation rates and amplify mutational biases. And yet we can evolve clonal genomes and not always observe the same mutation types. For example, in the study introduced above, Foster *et al*. noted that the type of transition mutations observed in mutator lines changed depending on both growth media and incubation temperature [29]. Why? Because mutagenic mechanisms do not operate consistently *ad infinitum*. Instead, the rates of genomic replication, the expression of its genes, and the exposure to toxic agents each vary depending on external triggers from the environment [55]. This means that genomic features associated with causing mutation can in many instances be described as having mutagenic potential, but this potential will change depending on the phase of the bacterial growth cycle and the presence of other stressors. Therefore, to gain a deeper understanding of which features are driving mutation within our genomes of interest, we must consider them in tandem with growth-associated mutagenesis [56] and stress-induced mutagenesis [57].

**Fig. 2.**
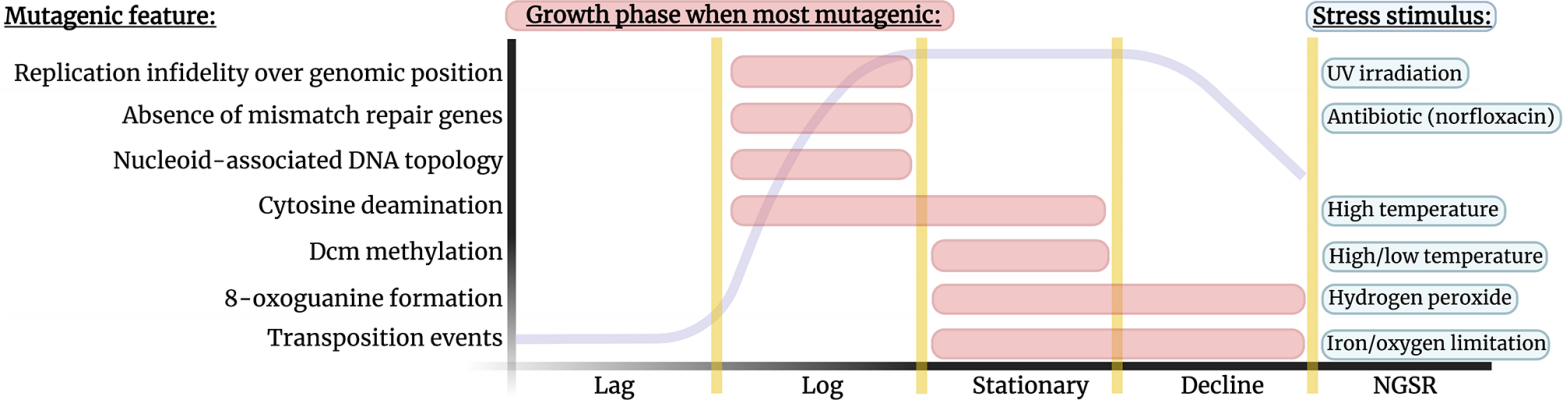
Mechanisms of mutation bias are affected by the bacterial growth cycle and other environmental stressors. A selection of mutagenic features (left) vary in their mutagenicity depending on the bacterial growth phase (heightened mutagenicity highlighted by red bars). The mutagenicity of each feature can additionally be amplified by non-general stress response (NGSR) triggers, as shown on the right. The effect of environment and growth phase on the listed features reflect studies performed on common model organisms, however there will be genomic backgrounds for which these trends do not apply. For example, while many bacteria most actively replicate during the logarithmic phase, the cyanobacteria *Synechococcus elongatus* performs intensive replication during the lag phase [165], meaning replication infidelity may be most mutagenic during an earlier phase. Full details of the publications informing this figure are provided in Table S1. Figure created with BioRender.com.

Some examples of growth phase-sensitive mutagenic mechanisms are shown in Fig. 2. The traits depicted in this figure reflect the literature and therefore are based on traits observed primarily in model laboratory species, especially *E. coli* (see Table S1, available in the online version of this article). Note therefore that there are genomic backgrounds for which some of these principles do not hold. In some cases, mutagenic mechanisms operate during all phases of growth, but become more mutagenic during a particular growth phase. For example, fluctuating replication fidelity may drive bias any time replication occurs, but this will be felt more keenly during exponential phase when replication occurs most frequently in most bacteria. In other cases, genomic features only become mutagenic in certain growth phases, whereas they can suppress mutation in others. For example, nucleoid-associated proteins that bind to DNA and alter its topology actively protect bound DNA from mutation during periods of stress and exposure to toxic agents [20, 58]. But multiple studies have found evidence that bound and superhelical DNA structures increase mutagenesis during replication, likely due to their interference with replicative machinery [20, 59, 60]. Similarly, Abundiz-Yañez *et al*. have recently found that in *B. subtilis*, functional diadenylate cyclases suppress mutagenesis in growing cells, but during nutrient stress of the stationary phase this trend is reversed and the cyclases instead promote mutation [56].

The time spent in each growth phase and the likelihood of encountering other stressors will fluctuate substantially depending on the colonized *in vivo* niche or the conditions of the laboratory experiment. Environments in clinical samples are more complex, with colonized organs such as the gut or the cystic fibrosis lung containing a myriad of heterogenous niches that vary from patient to patient [61, 62]. In contrast, lab-based systems provide more control over growth and stress factors. These factors will change depending on whether we use chemostats or turbidostats, rich or poor nutrient broths and agars, agitated or static cultures. Each choice will affect the generational times spent in each of the growth phases and the evolving population’s subjection to stress. Turbidostats for example can ensure that there is always an excess of nutrients facilitating exponential growth, whereas chemostats can prolong the stationary phase for many generations [63]. The stationary phase is associated with the general stress response, where gene expression changes to protect the cell [64] at the expense of increased mutation rate [65]. A key driver of stress-induced mutation during this phase is the upregulation of the sigma factor RpoS [66]. This protein increases mutation rate during stationary phase but can do so unevenly, promoting certain mutation types more than others (e.g. G:C → T:A rates increase but G:C → C:G rates do not [67]) and therefore drives mutation bias.

As nutrient-deficiency encountered by cells in the stationary phase drives stress-induced mutagenesis, environments that facilitate highly dense microbial populations experience greater nutrient limitation and thus exhibit a higher stress-induced mutational burden [68]. This suggests that growth rate and eventual population density should be positively correlated with mutation rate, but studies investigating both bacteria and yeast have found that they are instead often negatively correlated [69, 70]. This has been demonstrated by Krašovec *et al*. to be owed to a phenomenon termed density-associated mutation rate plasticity (DAMP) [70]. A highly mutagenic compound – oxidated guanine (8-oxo-G) – increases in abundance in the stationary phase (Fig. 2) but is combated by expression of MutT in *E. coli* [70], which prevents the misincorporation of 8-oxo-G opposite adenine in newly synthesized DNA strands [53]. Highly dense populations enjoy an increase in the number of 8-oxo-G scavengers and so exhibit lower mutagenesis from 8-oxo-G. Therefore there is a trade-off between mutations resulting from growth-associated population density and stress-induced mutations resulting from nutrient limitation [68].

Whether bacteria augment their environment during colonization of a new niche will also affect growth-associated and stress-induced mutagenesis. Agitating cultures coated with anti-adhesive compounds help to prevent the formation of biofilms [71], but biofilms are facilitated in static broth cultures [72] and on solid growth media. The formation of biofilms alters the local environmental niche for cells within the superstructure, within which the lack of nutrients and increase in toxin concentration can enable the SOS response and promote mutation [73]. In *E. coli *, this non-general stress response increases the expression of specialized DNA polymerases (Pol II, Pol IV and Pol V) that can perform translesion synthesis but are error prone [74]. A recent review by Joseph and Badrinarayanan summarized how translesion synthesis can promote mutational biases, as different mutation types were reported depending on the type of lesion and the error prone polymerase interacting with it [75]. Taken together, these reports highlight how simple changes to the experimental regime can have strong ramifications on which mutagenic mechanisms will be most active during evolution and which mutational biases they will promote.

In addition to variations of nutrients and spatial niches, bacteria are faced with selective challenges that can be highly threatening to population survival. These include the presence of toxins, which can trigger non-general stress responses and promote different types of mutation (Fig. 2). But perhaps a more notable mutagenic environmental trigger is the challenge of antibiotics, which is a common application in both clinical and laboratory strains. Antibiotics have long been known to cause stress that increases mutation rate in bacteria [76]. A study from Long *et al*. highlighted that certain antibiotics – in this case, norfloxacin – can also promote mutational biases, with G:C→T:A transversions becoming more frequent in *E. coli* with increasing dosage of the antibiotic [77]. Therefore, even the treatment method can itself alter bias and change the resultant mutational spectrum.

## The silent partner – when mutation bias can guide adaptation in the lab and the clinic

In evolving populations where standing genetic variation exists, adaptive alleles compete for dominance. Either the fittest of these genotypes reaches fixation (i.e. the genotype rises to a high frequency) through natural selection [78], or a population bottleneck reduces diversity such that chance becomes a stronger influencer [79]. Alternatively, multiple genotypes can persist and co-exist in the population [80–82], providing an opportunity for a secondary mutant descendent to evolve and achieve fixation [83]. Such competition between standing genetic variation provides opportunity for a ‘soft’ selective sweep, coined by authors Hermisson and Pennings [84]. The authors described soft sweeps as more likely to be observed when the potential fitness gain from mutations is modest, or, if the potential fitness gain is high, the mutational supply (or starting mutational diversity) is also high. In contrast, a ‘hard’ selective sweep involves the *de novo* arrival into the population of a single selectively advantageous allele that sweeps to dominance before a rival adaptive mutation appears [84]. In this latter ‘origin-fixation’ model [85], where the first adaptive mutation to occur will reach fixation, mutation bias can become a defining adaptive force [86, 87].

The context-dependency of mutation bias influence can be well illustrated by Cano *et al*. [10], who found that known biases are reflected in adaptive outcomes for three diverse species of microbes [10]. However, the authors also showed that using mutation bias as a predictive tool for adaptive evolution becomes less reliable as mutational supply and mutational target size increases [10]. This finding can be explained by re-visiting Hermisson and Penning’s earlier assertions [84]; as we increase the rate at which new mutations arrive in the population, or a larger pool of mutations become adaptive, we increase the chance of multiple adaptive genotypes co-existing in the population before one reaches fixation. This shifts the adaptive dynamics from facilitating a ‘hard’ to a ‘soft’ selective sweep, lessening the impact of mutation bias. Therefore, for mutation bias to be at its most influential, we search for instances when adaptive mutations occurring at biased rates can undergo a hard sweep to dominance.

**Fig. 3.**
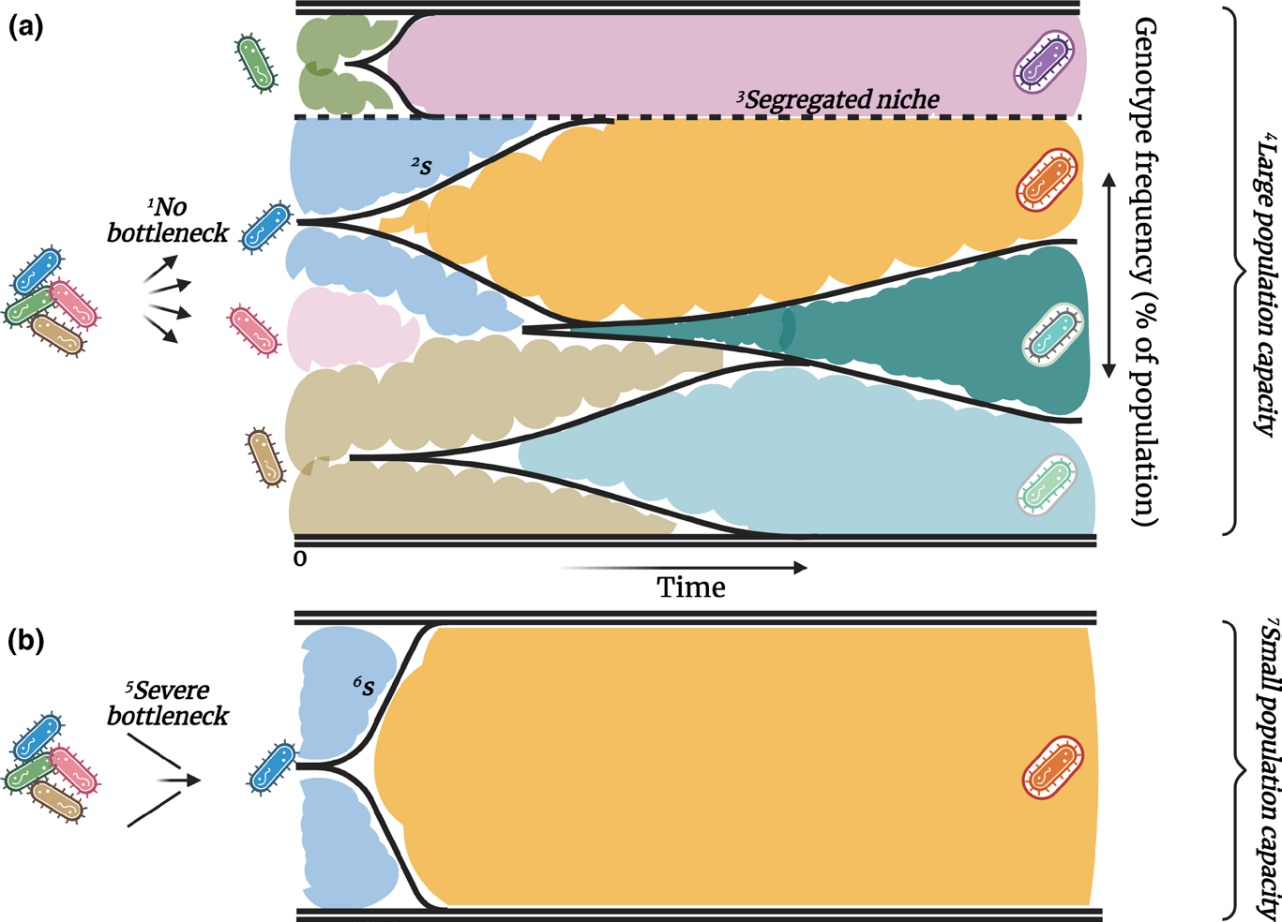
The role of mutation bias in determining adaptive outcomes is influenced by initial genetic diversity, the selective benefit (**s**) of novel mutations, the rate of mutation supply (μ), the number of isolated adaptive niches, and total population capacity. Shown in the two panels are toy Muller plots, which display how unique genotypes (individual colours) change in frequency (height of colour on *y* axis) over time (*x* axis). Colours that emerge after timepoint 0 represent evolved genotypes. Both scenarios show evolution to a novel environmental niche and include a genotype that exhibits potent mutation bias (blue), i.e. one specific adaptive genomic position exhibits a high relative localized mutation rate, which allows it to mutate rapidly (near timepoint 0) to a specific descendent genotype (orange). However, the orange genotype only dominates in one scenario. (**a**) Mutation bias does not play a dominant role in final genetic diversity: ^1^No bottlenecking means there are multiple genotypes initially evolving in the new niche. The biased genotype (blue) evolves rapidly, but the ^2^relative difference in fitness (**s**) between the descendent genotype (orange), its ancestor (blue), and other competing genotypes is not marked enough for it to rapidly dominate the population. This allows alternative adaptive mutants to appear from other genotypes, and for a member of the ancestor population to acquire a mutation (at rate μ) to a less common, but fitter genotype (teal) before the population goes extinct. The inability of the orange genotype to dominate is compounded by ^3^physically separated niches that allow alternate mutants to evolve in isolation (purple), ^4^and a large population capacity that increases the opportunity for multiple adaptive mutations to appear. (**b**) Mutation bias is the defining force in deciding the adaptive outcome: ^5^A severe bottleneck reduces starting genetic diversity to one genotype. Upon evolving at the biased position, ^6^the strong relative increase in fitness, compounded by ^7^a smaller population capacity without segregated niches, allows the evolved genotype to rapidly dominate the population pool before the wild-type genotype (blue) can mutate at rate μ to the more fit alternative genotype (teal in panel a). Scenario (b) is common in microbial evolution experiments, whereas clinical isolates will evolve in contexts somewhere between these two extremes. Figure created with BioRender.com.

As displayed in Fig. 3, for mutation bias to be at its most impactful we search for instances where (1) there is little to no standing adaptive genetic variation when selection is imposed, (2) an adaptive mutant can reach fixation before a competitor appears (i.e. mutation supply is low relative to the selective benefit of an adaptive mutation), (3) competition can take place across the population as sub-populations are not segregated in isolated niches. And that at least a portion of the genomic positions exhibiting biased mutation can prove adaptive. Determining how ubiquitous hard selective sweeps [criteria (1) and (2)] are in nature has been the subject of fierce debate [78, 88, 89], but is safe to say these criteria will not be satisfied under all adaptive scenarios. We can, however, find opportunities that meet these requirements during instances of microbial infection, where mutation bias has been reported [4, 90] and is sometimes imposed by the clinical treatment itself [91].

During infection, populations undergo severe bottlenecks that limit mutational diversity [92] as they pass through various defensive mechanisms including mucosal barriers [93] and organ filters [94]. A depletion of genetic diversity does not in itself guarantee a hard selective sweep (and by extension, a defining role for mutation bias). First, populations may begin as commensal bacteria and acquire renewed genetic diversity while evolving under purifying selection, before switching to a pathogenic phenotype [95]. Second, subsequent rounds of bottlenecks render populations susceptible to genetic drift [96], which can introduce more chance dependence into an adaptive outcome. Third, clinical isolates can evolve mutator phenotypes to cope with the selective burden imposed by host defences [97]. This increases the rate at which a beneficial (or deleterious) mutation will be realized, but also provides more opportunity for multiple adaptive genotypes to be simultaneously present in the population and compete for fixation [98]. However, modelling and experimental data has shown that when mutation supply is maintained at wild-type levels, the early arrival of an adaptive mutation can dominate and keep mutational diversity low [99]. Therefore, non-mutator, obligate pathogens undergoing limited numbers of bottlenecks can readily satisfy criteria (1) and (2). This means that microbial adaptation in the clinic can be highly guided by mutation bias, permitted genomic biases operate at adaptive positions [4, 90, 91].

While mutation bias in clinical isolates can be difficult to measure, a researcher can assume much greater control of adaptive variables in the laboratory. A recent study by Schenk *et al*. demonstrated the increased influence of bias when adaptive mutations can sweep to fixation before a rival mutation appears [requirement (2) above] [100]. They observed that in their *E. coli* strains, mutations resulting from structural variations (SVs) exhibited higher rates than single nucleotide polymorphisms (SNPs). This meant that SVs were biased to occur sooner than SNPs in their populations, despite SVs on average yielding lower fitness than SNP mutations [100]. The *E. coli* strains were propagated for approximately 500 generations in cultures facilitating either small or large population sizes. The authors observed that adaptive mutations in smaller populations were more likely to undergo a hard sweep to fixation. They also found that SVs were more likely to occur earlier in both population sizes, but the biased mutational class was more likely to reach fixation in smaller populations [100]. Owing to the number of cells, larger population sizes have a larger mutational supply (*μ*, Fig. 3) and it takes longer for an adaptive mutant to outcompete inferior genotypes and reach fixation (affecting *s*, Fig. 3). The study above demonstrates that even when a mutational class is biased to occur, the ability for this bias to determine adaptive outcomes is greatly influenced by the population dynamics (Fig. 3).

In some cases, efforts have been made to mimic clinical settings to uncover which variables are key to driving adaptation, including bottlenecks [100, 101]. Garoff *et al*., for example, found that soft selective sweeps were prevalent in evolving populations of *E. coli* when a large population was transferred through bottlenecks [101]. However, when smaller populations were transferred through bottlenecks, initially soft sweeps eventually ‘hardened’ [79] into hard selective sweeps as genetic diversity was sequentially lost [101]. Interestingly, the mutations fixed in the latter regime more closely matched mutations observed in clinical pathogens [101]. This provides another hint that in-host adaptation grants a context where mutation bias can be highly influential, as well as demonstrating that clinical evolutionary dynamics can be reflected in laboratory experiments.

It is common practice to initiate evolution experiments with clonal isolates [72, 102], which clears the runway for a hard selective sweep (Fig. 3b). The opportunity for a hard sweep is compounded if the initial microbial population is poorly adapted for the selective conditions, meaning there is ample opportunity for single-step mutations granting a large fitness increase at the onset of the experiment [102, 103]. Longer experimental designs provide opportunities for multiple sweeps as well as multiple rounds of bottlenecking [82, 104]; however, population bottlenecks can be avoided by performing longer experiments in a chemostat, which maintains population diversity [63]. In contrast, shorter experimental designs that isolate the first adaptive mutant to appear, such as when screening for a one-step mutation granting antibiotic resistance [105], can artificially enforce a ‘fixation event’ and further increase the prominence of mutation bias. Therefore, while heavily dependent on the experimental design, many laboratory experiment outcomes, i.e. those initiated with clonal populations evolved over short periods in a novel selective niche, are equipped to be strongly influenced by mutation bias.

## A hotspot in a haystack – utilizing genomic signatures to find potential bias

Determining whether bias is playing a role in the emergence of adaptive mutations requires detailed information about the mechanisms (Fig. 1) and environmental conditions (Fig. 2) driving the biases, as well as the evolutionary context to determine whether they are likely to be impactful (Fig. 3). But once armed with the mechanistic information we are primed to predict the genomic locations exhibiting bias. It has been observed that regions of biased mutation are not conserved even between close homologues [86, 106], and so identifying the mechanisms driving bias offer the best window into forming predictions of biased mutation. Model laboratory bacteria possess well-annotated genomes that contain accurate nucleotide sequence, gene locations and orientations, and a full complement of confirmed or predicted gene functions. As such they are excellent candidates to form mutational forecasts by searching genomic sequence for markers of bias.

**Fig. 4.**
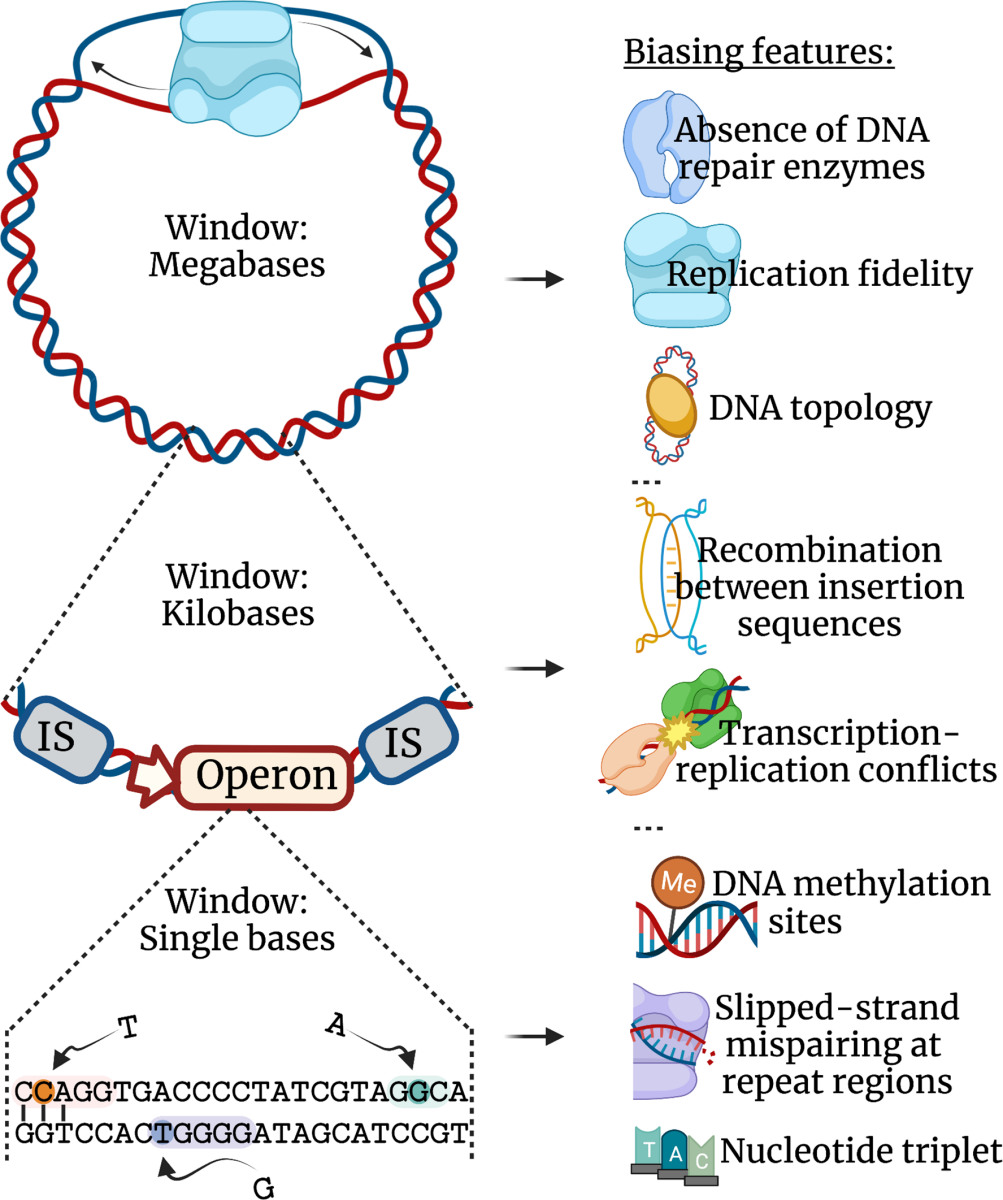
Genomic sequence data can be used to identify areas sensitive to exhibiting biased mutation. When analysing sequence data for mutagenic regions, features can be searched for at different scales, from bias operating across genomic regions to those operating on individual nucleotides. Examples of such features are listed on the right. For example, the ‘wave-like’ pattern of replication fidelity, which is correlated with genomic region (and likely affected by regions of DNA topology [166]) will fluctuate on the scale of the megabase. Therefore, an assembled genome with an annotated origin of replication can highlight genomic regions less sensitive to mutation. Likewise, the absence of coding regions for mismatch repair enzymes will cause a substitution bias (often a transition bias) throughout the entire genome, which can be identified through alignment searches. At the other end of the scale, if the window of interest is zoomed in to consider local nucleotide neighbourhoods, then small nucleotide motifs such as methylation target sites and homopolymer tracts can increase the rate and bias of mutation at single nucleotide positions. Such narrow investigations can be particularly insightful when examining the open reading frames and promoter regions of known adaptive gene targets. Figure created with BioRender.com.

Consider, for example, that we wished to predict whether a mutational hotspot at a specific nucleotide position existed within a locus of interest (Fig. 4). For diagnosing intra-locus mutation bias we can initially look extremely locally, narrowing our search window to just the nucleotides immediately flanking each focal base of interest, relying on the knowledge that the nucleotide triplet has a significant effect on base substitution rate [5, 29, 44]. Following this, if the base of interest is a cytosine in an enterobacterial genome, we can expand our window of interest slightly wider, scanning for a CCWGG (W is A or T) sequence flanking our focal nucleotide. This screens for a dcm methylation target site to see if the cytosine is more prone to deamination [107]. We next widen the window to a local neighbourhood of bases, inclusive of perhaps 50 nucleotides either side of our focal nucleotide. Here we can search for quasi-palindromic sequences that can facilitate intra-strand bonding and enable template-switching, which causes small insertions and deletions or single-base substitutions [108–111]. And we can search for homopolymeric tracts that either induce slippage events causing deletions [12, 29, 112] or a base substitution if the base of interest is a thymine preceded by a tract of guanine [17]. For these latter elements, a pipeline dedicated to recognizing hotspots using simple sequence repeats has already been developed [113].

If we wished to predict mutagenicity across an entire locus, we can widen our view to consider the genomic context of the target gene. Gene strandedness (encoding on the positive or negative strand) informs which strand of the gene is the coding and which the non-coding strand during transcription. It also informs which is the lagging strand and which is the leading strand during replication. Both of these elements have a bearing on strand-related biases across the locus. We may also consider the gene of interest’s proximity to recombination-causing flanking direct repeats, which can cause the locus to be rearranged or deleted [114–117], and their proximity to nucleoid-associated protein target sites, which determine a locus’ vulnerability to acquiring polymorphisms depending on the phase of growth [20, 58–60]. Finally, if we wish to establish broad genomic trends of bias, then we may look at the differential ‘wave-like’ patterns of mutation rates, where rates are often lower near the origin and terminus of replication and higher at intermediate distances [5, 24–26]. And we may evaluate the functionality or presence of mismatch repair genes [5, 118, 119].

Blokzilj *et al*. released a package in 2018 that uses human mutation data to expose trends in mutational patterns, which can then be used to infer the underlying mechanisms [120]. But there is also the opportunity to forecast mutation from a reference genomic sequence using our knowledge of mutational mechanisms (Fig. 4). These features can be incorporated into pipelines that allow us to identify sequence that is sensitive [121], or resilient [122], to mutation. And by doing so, we can enrich our understanding as to when mutation bias corroborates with selection to guide the adaptation of bacteria.

## Future work – experimental methods to recognize and quantify interacting forms of bias

Many of the mutagenic mechanisms driving mutation bias are now understood, yet we remain limited in our ability to identify likely sites of mutation bias that facilitate accurate forecasts of adaptation. One reason for this is that we must first acquire a generalizable and holistic understanding about how mutagenic mechanisms interact with one another. These mechanisms do not operate in isolation, but in tandem. For example, we know that methylated cytosines can become hotspots for mutagenesis following deamination [108], and there is an increased likelihood of deamination on the non-transcribed DNA strand [123]. Therefore, the two likely compound the mutagenicity of sites on which they both operate. Additionally, in human cancer cells, it is understood that both mismatch repair and transcription-coupled nucleotide excision repair drive strand-specific composition biases of mononucleotide repeats [124].

Features can therefore combine to raise the rates of mutation, but they can also counteract one another. Quasi-palindromic inverse repeats, for example, can cause a localized increase in mutation rate due to their interference with polymerase complexes [30]. Yet inverse repeats also allow single-stranded DNA to form intrastrand bonds that renders the DNA less exposed and susceptible to environmental damage [125]. In another example, a localized mutational hotspot may increase the mutation rate of a specific transversion mutation in an adaptive gene. In a wild-type genomic background this hotspot mutation may dominate the observed mutation pool following adaptation. However, placing this hotspot in a mutator context where transition mutations become more common can shift the observed spectrum to favour the realization of transitions, despite the hotspot’s continued presence [19]. We therefore must be able to recognize and quantify the interplay of genomic features to make predictors of mutational bias robust across genomic backgrounds.

**Fig. 5.**
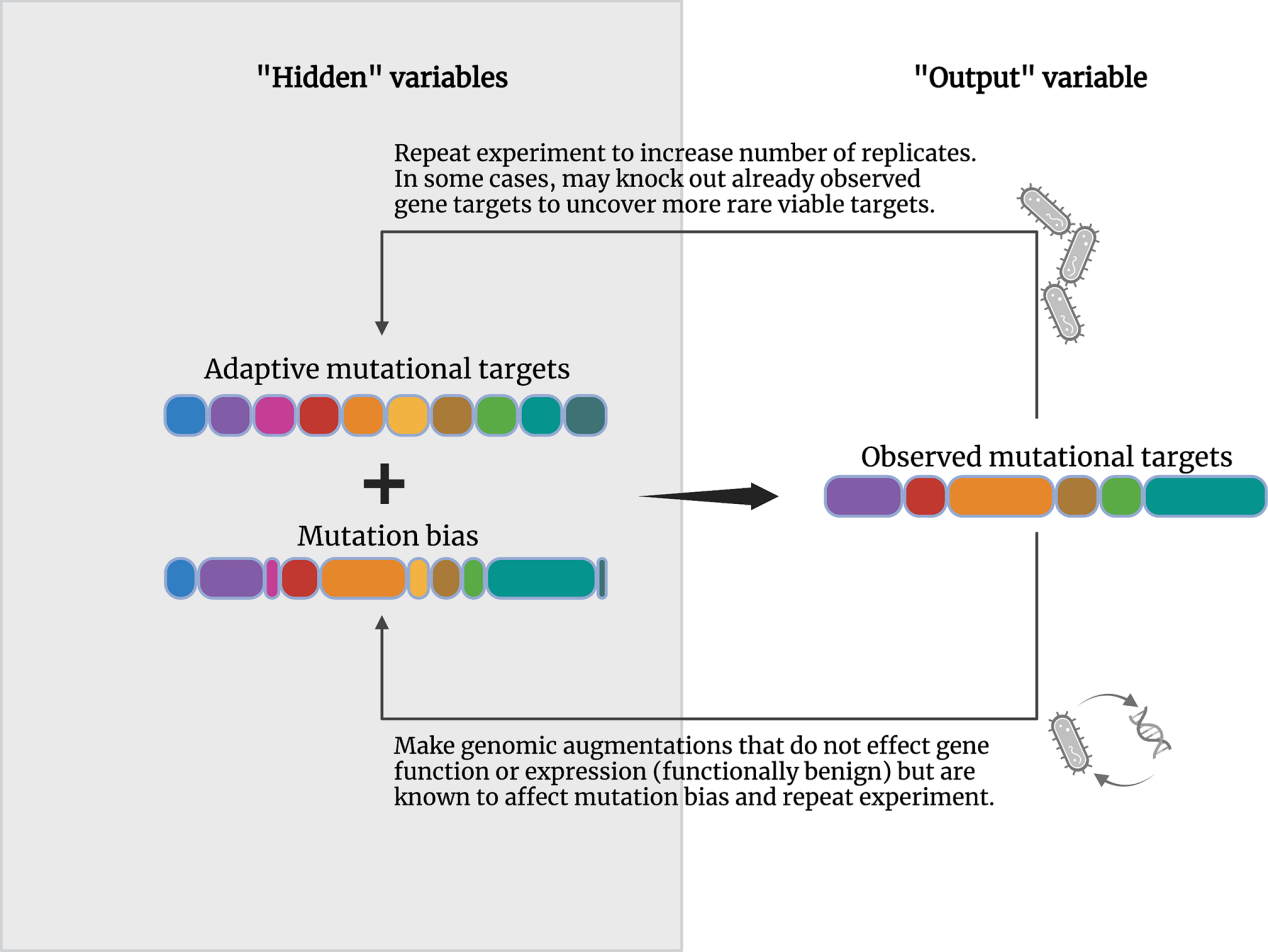
How to investigate mutation bias in laboratory experimental systems. Following a directed evolution experiment, sequencing techniques provide a researcher with a collection of observed mutational targets (i.e. the ‘output’ variable). However, the probability of observing a given mutation is defined by the number of possible mutations that can grant a phenotype under selection, and the mutational biases that operate on the positions within this pool (i.e., the ‘hidden’ variables). Experimental techniques that can elucidate the input of mutation bias and the full list of mutational targets are listed beside the lower and upper arrows. Figure created with BioRender.com.

So how can we recognize new forms of bias? When we evolve isolates in the laboratory or sequence evolved isolates from clinical samples, we rarely obtain a complete pool of the mutations that can facilitate a phenotype. Instead we observe a sample from the potential pool of mutations that can offer a phenotype (Fig. 5). The result of this is that we can readily observe the effect of selection, i.e. the enrichment of a particular mutation, but we are unable to recognize if there has been any input from mutation bias. This is because without knowing the complete pool of adaptive mutational targets, how can we know if the mutations we observe represent the full pool, or are just the mutations that are biased to occur, or are just a sub-set observed simply through chance?

Elucidating biases at play from the ‘bottom-up’, i.e. without any *a priori* knowledge of operating biases, will be crucial for uncovering and quantifying the potency of novel bias-driving interactions. In evolution experiments, biases in mutation during adaptation often manifest as instances of parallel or repeatable evolution [86, 87, 106]. Parallel evolution describes occasions where independently evolving populations acquire the same mutation (this makes the evolutionary outcome repeatable, hence why the terms are sometimes used interchangeably). In an experimental context, this involves repeating an identical experiment using clonal samples and repeatedly observing the same mutation or subset of mutations following adaptation [86, 87, 106]. Observations of parallel evolution are not always owed to mutation bias [126]; selection can act on a very low number of positions [127] and therefore a limited mutational target size makes some degree of parallelism inevitable. However, sometimes parallel evolution can be driven by a mutational hotspot [128], where a given mutation occurs at a higher rate than elsewhere. This means that while a pool of mutations are possible, we repeatedly observe mutations fixed at the hotspot site due to a strong mutation bias [86]. Such instances of parallelism are fine targets for using experimental methods to investigate agents of mutation bias (Fig. 5).

## Future work – does natural selection enforce, remove or mostly ignore agents of bias?

An alternative way to anticipate regions of the genome that will be more mutable than others is to consider the role played by natural selection. As most new mutations are deleterious [129], high mutation rates across the genome will be unfavourable to most organisms. The necessary suppression or control of mutation rates explains the ubiquitous evolution of DNA repair enzymes [46]; or the evolution of alternative DNA repair pathways for those organisms that did not evolve mismatch repair [130, 131]. However, there may be more nuance to the role of selection than enforcing non-discriminatory DNA repair enzymes. In a recent study looking at the flowering plant *Arabidopsis thaliana*, Monroe *et al*. reported that mutational biases in the genome reflect mutations fixed in natural evolving populations [122]. They observed that gene bodies and essential genes, which are better protected by epigenomic modifiers such as methylation and histone modifications, exhibit lower mutation rates relative to non-essential loci and intergenic regions [122]. Critics of this work have identified that many of the called mutations in this dataset are products of sequencing errors, which are enriched to occur in regions of perceived higher mutability [132, 133]. However, if the findings remain genuine despite these errors – as the authors argue [134] – this work suggests that selection can suppress mutation rates unequally throughout the genome.

If genuine, these observations can be explained by a theory that is likewise applicable to microbes, proposed by Lynch [135]. Lynch’s drift-barrier hypothesis posits that purifying selection (which removes deleterious mutations from a population) works to lower mutation rates, but this is counteracted by the random percolations associated with genetic drift [135, 136]. While this hypothesis does not allow for mutation rates to be specifically reduced for individual genes, if the length of the sequence affected is large enough (i.e. if we consider all essential genes and gene bodies together), selection may be able to drive mutation down over certain genomic regions [122]. Essential genes and others subject to strong purifying selection (meaning that a mutation is almost always strongly deleterious) will therefore be more likely to evolve lower mutation rates. Therefore, we might predict that loci outside of these regions will exhibit relatively higher mutation rates.

A more controversial argument is that selection actively enforces higher mutation rates when these will be beneficial. Above, we discussed growth-associated and stress-associated mutagenesis that result in higher mutation rates. There is an argument that the higher mutation rates might be an advantageous part of the stress response, as they act to elevate rates when the cell is stressed so that mutation can be utilized for evolutionary escape. MacLean *et al*. [57] have argued that stress-induced mutagenesis is likely owed to a combination of factors [57], including genetic drift and positive selection for the reduction of damage from stressors. This positive selection indirectly also selects for increased mutation rate as the same proteins determine both traits [57]. The capacity for mutagenic mechanisms to hitchhike alongside positively selected traits is known as second-order selection [137]. This phenomenon also explains how mutator genotypes lacking mismatch repair can be indirectly selected for under conditions where mutation is necessary for cell survival, as the mutator genotypes are more likely to produce an adaptive mutation [138]. A recent study by Sane *et al*. has also suggested that the change in mutational bias in mutator genotypes provides access mutational types that appear less often in wild-type backgrounds [11]. They argue that these under-sampled mutation types are more likely to be adaptive [11], therefore a change in mutation bias can be indirectly supported by a second-order selection effect. However, direct selection for higher mutation rates or changes in mutation bias have yet to be conclusively demonstrated (although they have recently been implicated in yeast [69]).

Aside from stress-associated mutation and mutators, which affect mutation rates throughout the genome, others have argued that DNA sequence is sometimes selectively primed to mutate at specific positions. Mutable DNA sequences capable of targeted mutation include homopolymer or tandem repeats of nucleotides, which readily undergo reversible insertions and deletions at a high rate [113]. These have been described to readily occur in genes termed ‘contingency loci’ by Moxon *et al*. [139], who argued that higher rates of mutation are found in genes capable of introducing phenotypic variation in changing environments, such as those encountered within a host [140]. When placed in a promoter sequence or open reading frame, a single insertion or deletion event can remove the in-frame expression of the contingency locus. This mutation may be useful initially but later cease to be useful, as could be the case in a fluctuating environment. But the elevated indel rate at the tract allows the sequence to later revert the mutation and recover its wild-type genotype and phenotype. More broadly, the process of bacterial populations rapidly generating reversible phenotypic diversity is known as phase variation. Pathogens have been noted to employ phase variation to cope with bottlenecks and the loss of genetic diversity when infecting hosts, and phase-variable loci are typically found in cell-surface genes such as adhesins and pili [141, 142]. Phase variation does not require mutagenesis from contingency loci, as changes in gene expression can be achieved instead through epigenomic modifications [143]. However, several studies have demonstrated that localized mutagenic positions can be favoured under selection [16, 144–146]. These include a recent study that detailed how *B. fragilis* utilizes reversible DNA inversions to control the expression of capsular polysaccharides as a means to cope with periods of inflammation [147]. However, whether these positions have been maintained by selection in microbial pathogens is less clear [140].

Finally, it is worth considering the mutagenic mechanisms that are ‘less visible’ to selection. Many drivers of mutation bias are influenced by the local nucleotide context (Fig. 1). These include homopolymer tracts that cause slipped-strand mispairing [12], methylation recognition sites that cause cytosine deamination [108] inverse repeats that result in hairpin formation and template switching [30], and nucleotide triplets that affect mismatch repair efficiency [29]. As these features rely on short tracts of nucleotides, changing a handful or sometimes a single nucleotide can affect their mutation rates drastically [86]. Further, these base-pair substitutions can be synonymous [86]. While synonymous mutations are now appreciated to be capable of causing changes to fitness [148–151], they are typically under weaker selection than non-synonymous changes [152]. As such, nucleotide neighbourhoods driving divergences in localized mutation biases may not require purifying or positive selection but may appear and degrade in a population simply through synonymous changes via the process of genetic drift.

Whether selection suppresses, elevates, or is mostly ineffectual in governing mutational biases, many questions remain unanswered. For example, there is some empirical evidence that mutation hotspots might be more common in certain types of genes. Multiple experimental evolution studies have found mutation hotspots within histidine kinases at the nexus of regulatory pathways [18, 86], or in other downstream effector genes [16, 153]. Such genes often illicit significant changes to phenotypes. But do we see mutational hotspots in these sequences because of their adaptive capacity, or simply because such hotspots are easier to find due to the phenotypic changes they provoke? It is likely that the identification of these hotspots is owed to the latter explanation. If so, then hotspots are not found in these genes because of positive selection and yet they are found relatively often. This suggests that hotspots may be fairly common, and thus must be able to evade purifying selection at least for a time. Therefore, localized differences in mutational biases may be found throughout the genome and be more widespread than current experimental evolution data suggests.

## Conclusion

There has been a growing interest in whether we can improve our ability to predict evolution. A deeper understanding of mutation biases and their role in defining adaptation moves us closer to this goal. In this review, we have discussed the causes and consequences of mutation biases on bacterial adaptation in the lab and in a clinical setting. We have highlighted the immediate challenge that lies ahead, which is bringing together our existing knowledge of how mechanisms of mutation bias work in isolation and use empirical investigation to unravel how they interplay. Efforts have been made to collate mutational data from experimental evolution studies [154]. Such pursuits have the potential to reveal trends that allow for the identification of mutational hotspots and other signals of mutation bias occurring in adaptive genes of interest.

We must also begin to consider the genomic context of mutation bias – for example, we cannot assume an identical mutagenic mechanism is driving mutational bias just because it is facilitating a common outcome [155]. Therefore, we must also gain evidence to define mechanisms of bias across different genomic contexts and within different genomic backgrounds. For example, mutation accumulation experiments in the Lynch laboratory identified that mutation rates are lower toward the origin and terminus of replication in *Pseudomonas fluorescens* [5]. But a mechanism explaining this effect was only implicated when they later performed a similar experiment that compared mutation rates between the core and secondary chromosomes in *Vibrio* species [25]. This demonstrates the power of using alternative bacterial species to both test the robustness and elucidate the mechanism of biasing features in bacterial genomes.

Together, this knowledge may provide the inputs necessary for the ultimate challenge of creating pipelines that will be able to determine mutability at a given site based on information from surrounding genetic features and genomic details. To achieve these ambitious goals will require a collaborative effort between empirical and bioinformatic research groups. It will also require high-throughput approaches to mutation detection, which will increase the likelihood of effectively elucidating the complete pool of mutational adaptive targets – something we are yet to achieve at the necessary scale [156]. Open sharing of data (e.g. [154]) will also be vital for progress to allow bioinformatic approaches to be implemented in a way that will be informative generally across bacteria. However, given the rapid progress that this field has made in recent years, and the growing interests of research groups in achieving predictive power of evolutionary outcomes, we are excited to follow the advances and innovative approaches to the challenges discussed.

## Supplementary Data

Supplementary material 1
