# Supplementary material for: Mutation bias and adaptation in bacteria
Source: Microbiology (Reading). 2023 Nov 9;169(11):001404. doi: 10.1099/mic.0.001404 (PMC10710837; doi:10.1099/mic.0.001404)
Supplement: Supplementary material 1 [file mic-169-1404-s001.pdf]

Supplementary Table S1. Details of publications demonstrating growth phase and other environmental stressor's effect on mutagenic mechanisms.

**Mutagenic feature:**	**Growth phase when most active:**	**Demonstrated in:**	**Ref:**	**NGSR stimulus:**	**Demonstrated in:**	**Ref:**
Replication infidelity over genomic position	Log (replication predominantly occurs during log phase)	*P. fluorescens (ATCC948), V. fischeri, V. cholerae, E. coli*	[1] [2] [3]	Increased dNTP pools associated with wave-like mutation rates via genomic modifications. However, ultra-violet irradiation has been shown to increase dNTP pools and mutagenesis.	*V. fischeri* and *V. cholerae, E. coli;* also demonstrated in yeast.	[2] [4] [5]
Absence of mismatch repair genes	Log (MMR primarily active during DNA replication and recombination)	*E. coli*	[6]	Antibiotic (norfloxacin)	*E. coli (K-12)* – increases mutation rate in MMR- strains, and increases transversion bias in MMR+ strains	[7]
Nucleoid-associated DNA topology	Log (Suggested by: argued to interfere with DNA repair proteins active in log phase; and inference that DNA replication becomes inaccurate in regions of high superhelical density)	*B. subtilis, Escherichia (various species), Shigella (various species)*	[8] [9]			
Cytosine deamination	Log and stationary (the periods of high replication and transcription)		[10] [11] [12]	High temperature	Ex vivo	[13]
Dcm methylation	Stationary (suggested by: M9 minimal media + 96-hour samples having more methylation; also, strong expression control of genes by methylation in stationary phase over log phase observed).	*E. coli (strains SC419 and SC452, also various from ECOR)*	[14] [15] [16] [17]	Temperature	*E. coli* (high temp: strain *SC419,* low temp: strain *SC452*)	[14]
8-oxoguanine formation	Stationary onward (Reactive oxygen species accumulate during growth, resulting in increased amounts of 8-oxo-guanine that continues to cause mispairing in non-dividing cells persisting with	*E. coli*	[18] [19]	Hydrogen peroxide (this engages the general stress response but can also change the expression of other stress response pathways, e.g. OxyR and heat shock proteins).	*E. coli, S, enterica*	[20] [18] [21] [22] [23]

	transcription later in the growth cycle.)					
Transposition events	Stationary and death	*P. putida (Tn4652)*	[24]	Iron/oxygen deficiency	*E. coli (IS150)*	[25] [26]

References:

1. **Long H, Sung W, Miller SF, Ackerman MS, Doak TG, *et al.*** Mutation rate, spectrum, topology, and context-dependency in the DNA mismatch repair-deficient Pseudomonas fluorescens ATCC948. *Genome Biol Evol* 2014;7:262–271.

2. **Dillon MM, Sung W, Lynch M**. Periodic Variation of Mutation Rates in Bacterial Genomes. *mBio* 2018;9:1–15.

3. **Zhang X, Zhang X, Zhang X, Liao Y, Song L, *et al.*** Spatial Vulnerabilities of the Escherichia coli Genome. *Genetics* 2018;210:547–558.

4. **Watt DL, Buckland RJ, Lujan SA, Kunkel TA, Chabes A**. Genome-wide analysis of the specificity and mechanisms of replication infidelity driven by imbalanced dNTP pools. *Nucleic Acids Res* 2015;44:1669–1680.

5. **Gon S, Napolitano R, Rocha W, Coulon S, Fuchs RP**. Increase in dNTP pool size during the DNA damage response plays a key role in spontaneous and induced-mutagenesis in Escherichia coli. *Proc Natl Acad Sci U S A* 2011;108:19311–19316.

6. **Li G-M**. Mechanisms and functions of DNA mismatch repair. *Cell Res* 2008;18:85–98.

7. **Long H, Miller SF, Strauss C, Zhao C, Cheng L, *et al.*** Antibiotic treatment enhances the genome-wide mutation rate of target cells. *Proc Natl Acad Sci* 2016;113:E2498–E2505.

8. **Warnecke T, Supek F, Lehner B**. Nucleoid-Associated Proteins Affect Mutation Dynamics in E. coli in a Growth Phase-Specific Manner. *PLoS Comput Biol*;8. Epub ahead of print 2012. DOI: 10.1371/journal.pcbi.1002846.

9. **Niccum BA, Lee H, Mohammedismail W, Tang H**. The Symmetrical Wave Pattern of Base-Pair Substitution Rates across the Escherichia coli Chromosome Has Multiple Causes. *mBio*;10.

10. **Bhagwat AS, Hao W, Townes JP, Lee H, Tang H, *et al.*** Strand-biased cytosine deamination at the replication fork causes cytosine to thymine mutations in Escherichia coli. *Proc Natl Acad Sci U S A* 2016;113:2176–2181.

11. **Francino MP, Ochman H**. Deamination as the Basis of Strand-Asymmetric Evolution in Transcribed Escherichia coli Sequences. *Mol Biol Evol* 2001;18:1147–1150.

12. **Beletskii A, Bhagwat AS**. Transcription-induced mutations: Increase in C to T mutations in the nontranscribed strand during transcription in Escherichia coli. *Proc Natl Acad Sci U S A* 1996;93:13919–13924.

13. **Lewis CA, Crayle J, Zhou S, Swanstrom R, Wolfenden R**. Cytosine deamination and the precipitous decline of spontaneous mutation during Earth's history. *Proc Natl Acad Sci* 2016;113:8194–8199.

14. **Breckell GL, Silander OK**. Growth condition-dependent differences in methylation imply transiently differentiated DNA methylation states in Escherichia coli. *G3 GenesGenomesGenetics* 2023;13:jkac310.

15. **Cherry JL**. Methylation-Induced Hypermutation in Natural Populations of Bacteria. *J Bacteriol* 2018;200:e00371-18.

16. **Militello KT, Simon RD, Qureshi M, Maines R, Van Horne ML, *et al.*** Conservation of Dcm-mediated Cytosine DNA Methylation in Escherichia coli. *FEMS Microbiol Lett* 2012;328:78–85.

17. **Kahramanoglou C, Prieto AI, Khedkar S, Haase B, Gupta A, *et al.*** Genomics of DNA cytosine methylation in Escherichia coli reveals its role in stationary phase transcription. *Nat Commun* 2012;3:886.

18. **Alhama J, Ruiz-Laguna J, Rodriguez-Ariza A, Toribio F, López-Barea J, *et al.*** Formation of 8-oxoguanine in cellular DNA of Escherichia coli strains defective in different antioxidant defences. *Mutagenesis* 1998;13:589–594.

19. **Sekowska A, Wendel S, Fischer EC, Nørholm MHH**. Generation of mutation hotspots in ageing bacterial colonies. *Sci Rep* 2016;6:4–10.

20. **Battesti A, Majdalani N, Gottesman S**. The RpoS-Mediated General Stress Response in Escherichia coli. *Annu Rev Microbiol* 2011;65:189.

21. **Storz G, Spiro S**. Sensing and Responding to Reactive Oxygen and Nitrogen Species. In: *Bacterial Stress Responses*. John Wiley & Sons, Ltd. pp. 157–173.

22. **Fulda S, Gorman AM, Hori O, Samali A**. Cellular Stress Responses: Cell Survival and Cell Death. *Int J Cell Biol* 2010;2010:214074.

23. **Fasnacht M, Polacek N**. Oxidative Stress in Bacteria and the Central Dogma of Molecular Biology. *Front Mol Biosci*;8. https://www.frontiersin.org/articles/10.3389/fmolb.2021.671037 (2021, accessed 22 March 2023).

24. **Ilves H, Hõrak R, Kivisaar M**. Involvement of ςS in Starvation-Induced Transposition of Pseudomonas putidaTransposon Tn4652. *J Bacteriol* 2001;183:5445–5448.

25. **Maharjan RP, Ferenci T**. A shifting mutational landscape in 6 nutritional states: Stress-induced mutagenesis as a series of distinct stress input–mutation output relationships. *PLoS Biol*;15. Epub ahead of print June 2017. DOI: 10.1371/journal.pbio.2001477.

26. **Shewaramani S, Finn TJ, Leahy SC, Kassen R, Rainey PB, *et al.*** Anaerobically Grown Escherichia coli Has an Enhanced Mutation Rate and Distinct Mutational Spectra. *PLOS Genet* 2017;13:e1006570.
